# *Porcine circovirus 3* (PCV-3) variability: Is it in the virus or in the classification criteria?

**DOI:** 10.1186/s12985-023-01984-6

**Published:** 2023-02-09

**Authors:** Giovanni Franzo, Joaquim Segalés

**Affiliations:** 1 Department of Animal Medicine, Production and Health (MAPS), Padua University, 35020 Legnaro, Italy; 2 Departament de Sanitat i Anatomia Animals, Facultat de Veterinària, Universitat Autònoma de Barcelona, 08193 Bellaterra, Spain; 3 Unitat Mixta d’Investigació IRTA-UAB en Sanitat Animal, Centre de Recerca en Sanitat Animal (CReSA), Campus de la Universitat Autònoma de Barcelona (UAB), 08193 Bellaterra, Spain; 4 OIE Collaborating Centre for the Research and Control of Emerging and Re-Emerging Swine Diseases in Europe (IRTA-CReSA), 08193 Bellaterra, Spain

**Keywords:** *Porcine circovirus 3* (PCV-3), Genotyping, Classification, Standardized criteria, Variability

## Abstract

The continuous discovery of new viruses during the last decades has increased the need for new classification approaches and rules. Currently, the International Committee on Taxonomy of Viruses classifies viruses up to the species level. However, because of the higher variability of most of these infectious agents, a below-species categorization is often required for proper epidemiological investigations. Unfortunately, variable criteria are typically proposed by different research groups, leading to misleading and poorly reproducible results. This scenario occurred for the recently identified *Porcine circovirus 3*. Although genotype definition standards had been defined by a group of experts in the field, recent articles have been published introducing new genotypes, whose classification rules are not reported. We therefore would like to stress the usefulness of defining and maintaining a common language to allow proper results comparison among groups. We consider the consensus opinion of a heterogeneous expert team as the most valuable approach. Nevertheless, if other approaches are proposed, the disclosure of the criteria and the comparison with previous literature should be deemed mandatory to allow effective results reproducibility, interpretation and sharing.

## Background

The last decades have been featured by the discovery of an astonishing number of new viruses, often through molecular techniques only. The need for organizing such an amazing amount of new data has prompted an intensive classification or re-classification and naming efforts, which are usually based on the increasing knowledge on genome sequences and their similarity rather than on phenotypic or clinical features.

According to the International Committee on Taxonomy of Viruses (ICTV, https://ictv.global/), viruses are classified up to the species level. However, because of the remarkable variability and high evolutionary rates of several viral species, the “species-level” resolution often fails to properly describe the epidemiological and biological features of most viruses infecting humans, animals or plants. For this reason, different proposals of sub-species classification have been suggested for most viruses, and porcine circoviruses (PCV) are not an exception.

The most well-known PCV, *Porcine circovirus 2* (PCV-2), was subjected to further subclassifications (clades, genogroups, genotypes) since the first years after its identification [1]. Although conflicting criteria and nomenclature were initially published [2–5], a consensus on sub-species (genotypes) classification criteria and names was reached among researchers over time [4]. Moreover, the global convergence at the international level to specific criteria, including regular updates [6, 7], allowed the establishment of a common language among people working in the field.

A comparable scenario emerged with the identification of *Porcine circovirus 3* (PCV-3) [8]. Based on the positive PCV-2 experience, sub-species classification criteria and names were proposed by a team of circovirus experts from several research groups located in different parts of the world [9]. Briefly, the following criteria were suggested and validated to define a PCV-3 genotype: maximum within-genotype genetic distance of 3% at the complete genome and 6% at the ORF2 levels and bootstrap support or posterior probability in the phylogenetic tree higher than 90%. Finally, it was suggested to formally accept a genotype only if at least five sequences are available in gene databases, to avoid the risk of defining poor-quality sequences or extremely low-fitness strains as separate genotypes. According to these criteria, consistent results were obtained using different analysis methods and datasets, and a single genotype was proposed (named as PCV-3a). Although sub-clusters could be identified within PCV-3a genotype, the bootstrap support was often low and inconsistencies among different datasets and analysis methods were observed, suggesting caution in the recognition of additional genotypes. To facilitate and standardize further studies, a reference dataset was provided in the original publication [9].

## Main text

The efforts for unifying a classification at a viral sub-species level are important to offer a common language and facilitate comparisons among epidemiological studies at regional, national and worldwide levels. Therefore, the objective of this Comment is to get the attention of the international scientific community regarding the convenience of keeping clear-cut classification criteria and using them in subsequent studies, at least until potential new well-substantiated ones may replace the original criteria. More specifically, this Comment wants to address the PCV-3 classification used in some recently published studies [10–12].

These studies [10–12] report a variable number of PCV-3 genotypes, with different names from those that have been previously proposed [9]. These works mention up to three different genotypes (PCV-3a, 3b and 3c). Since the existing data to date pointed out to one single genotype for PCV-3 (PCV-3a) [9], the description of novel genotypes is of worldwide interest. As an example, we would like to comment on the case of *Epidemiological and genetic characteristics of Porcine circovirus 3 in 15 provinces and municipalities of China between 2016 and 2020* [10]. When the so-called genotype (PCV-3a, 3b and 3c) sequences are aligned, all their strains were closely related to the PCV-3a ones previously reported [9]. Moreover, the maximum genetic distance among these reported ORF2 sequences was 0.0388 (3.8%), lower than the threshold suggested for a new genotype definition. Therefore, based on the previously proposed criteria, all their sequences would be PCV-3a. Noteworthy, these authors [10] did not provide a comprehensive definition of genotyping criteria in their article, which further confuses the nomenclature and makes it difficult to compare different studies. This scenario clearly complicates the understanding of PCV-3 epidemiology and can be extremely misleading for researchers and, especially, for field veterinarians, farmers and commercial companies also.

While we assume that, being not officially recognized, every sub-species classification scheme should be scientifically scrutinized, we consider counterproductive to introduce new schemes in absence of reasonable biological or epidemiological reasons to update or replace pre-existing criteria. Additionally, when new genotypes are reported, the criteria for such classification are typically absent and no comparison with previous literature is performed, making these approaches arbitrary and poorly reproducible.

## Conclusion

Based on these considerations, we believed it opportune to stress the need to comply with shared standards for PCV-3 sub-species classification criteria, to allow the creation of a common language among researchers and people working in the field. Although not formally recognized by the ICTV, the below-species level classification represents an invaluable tool for the understanding of viral infection epidemiology and, potentially, for their control. Since no universal rules for such sub-species level classification have been established for viruses, we consider the consensus opinion of a heterogeneous expert team the most valuable approach. Nevertheless, if other approaches are proposed, the disclosure of the criteria and the comparison with previous literature should be deemed mandatory to allow results comparison and reproducibility.
